# Emergency Department Diagnosis of Idiopathic Pneumoparotitis with Cervicofacial Subcutaneous Emphysema in a Pediatric Patient

**DOI:** 10.5811/cpcem.2017.7.34879

**Published:** 2017-11-03

**Authors:** Khai Pin Lee, Vigil James, Gene Y. Ong

**Affiliations:** KK Women’s and Children’s Hospital, Department of Pediatric Emergency Medicine, Singapore

## Abstract

Idiopathic pediatric pneumoparotitis, being rare, is often misdiagnosed in acute care settings, resulting in inappropriate initial management and emergency department (ED) disposition. We report the case of a previously well 11-year-old boy who presented to our ED with acute left cheek swelling and pain. He was diagnosed with pneumoparotitis with cervicofacial subcutaneous emphysema with the aid of point-of-care ultrasound (POCUS) and radiographs. Despite appropriate initial ED and inpatient management, he developed bilateral involvement and pneumomediastinum. After 72 hours, his condition improved and he was discharged well after five days of hospitalization. This case report highlights the use of POCUS and radiographs to facilitate an early diagnosis and appropriate ED disposition.

## INTRODUCTION

Pneumoparotitis is a rare cause of painful parotid gland swelling and occurs when air passes through the parotid (Stenson) duct into the parotid gland.^1^ Subcutaneous emphysema can occur from an extension of the air leak from the affected parotid acini to the surrounding cervicofacial subcutaneous tissues.^1^,^2^ This can occur from any sudden increase in upper airway pressure facilitating transmission of air into the tissue planes. Underlying mechanisms from published case reports include unintentional orofacial trauma, severe bouts of coughing or sneezing, drug-snorting, playing a wind instrument, retching or vomiting, straining due to constipation, and repeated Valsalva maneuver performed by adolescents with psychological problems.^1^–^3^ It can also occur as a result of oropharyngeal procedures such as dental surgery and tonsillectomy, positive pressure ventilation after traumatic intubations, or any unintentional intra-oral trauma.^4^,^5^ Published literature on idiopathic or spontaneous pneumoparotitis with cervicofacial subcutaneous emphysema in children is sparse. Pneumoparotitis is often misdiagnosed in the emergency department (ED), resulting in inappropriate initial management and disposition.^3^

## CASE REPORT

A previously well, fully immunised, non-asthmatic 11-year-old boy presented to our pediatric ED with an acute history of left-sided facial swelling for six hours. There was severe pain, which worsened on mouth-opening and during mastication. He had no associated fever, cough, sneezing, vomiting, dysphagia or breathing difficulty. Significantly, there was no history of provocation such as recent oral trauma, dental procedures or oropharyngeal surgeries, blowing a balloon, playing a wind instrument, breath-holding, constipation or drug use. Given his parotid swelling and the possibility of mumps, he was initially put in an isolation consultation room by the triage nurses.

**Image 1:**
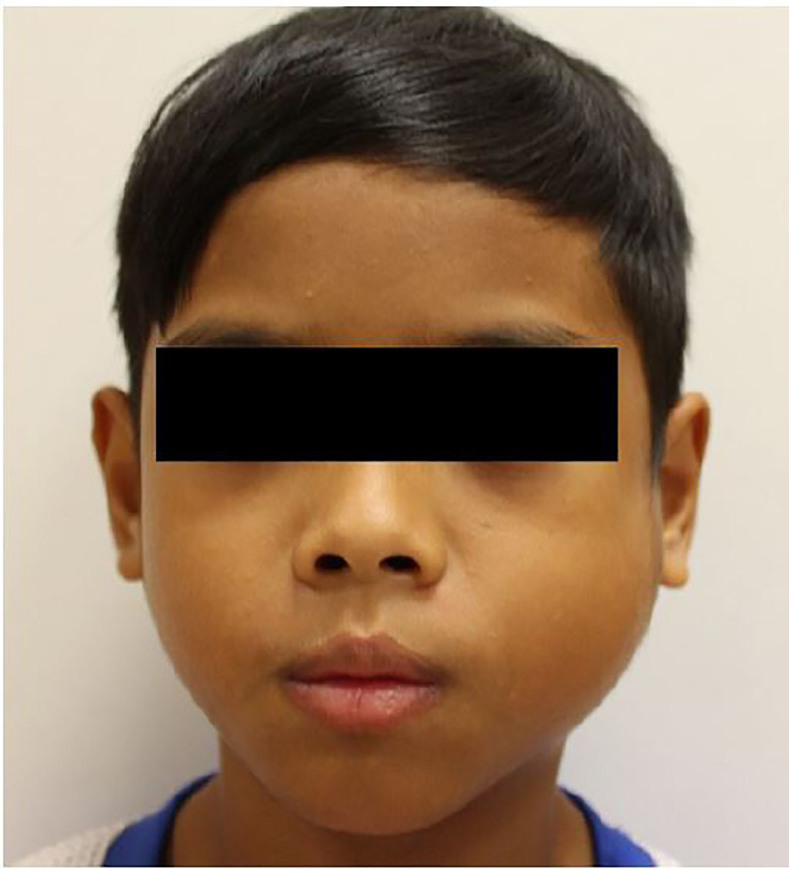
Patient with left-sided cheek swelling that elevated the pinna

On examination, the patient was stable with the following vital signs: heart rate 72 beats per minute, respiratory rate 20 per minute, blood pressure 118/60 mmHg, saturation oxygen 100% on room air, and temperature of 37.5 degree Celsius. He was alert and active with no evidence of respiratory distress. There was a tender, soft 7.5 cm × 7.5 cm ovoid pre-auricular swelling involving the left cheek, which elevated the left pinna (Image 1).

The ability to open his mouth was limited due to pain. Subcutaneous crepitations were elicited over the swelling and over the upper part of the left side of the neck. Intra-oral examination revealed normal dentition and throat anatomy, no swelling on the floor of the mouth, and no evidence of trauma. Otoscopic examination of his ears was normal and his neck was supple with full range of motion. Systemic examination revealed equal air entry in bilateral lung fields with no crackles or wheeze, and normal cardiac and abdominal examinations.

**Image 2:**
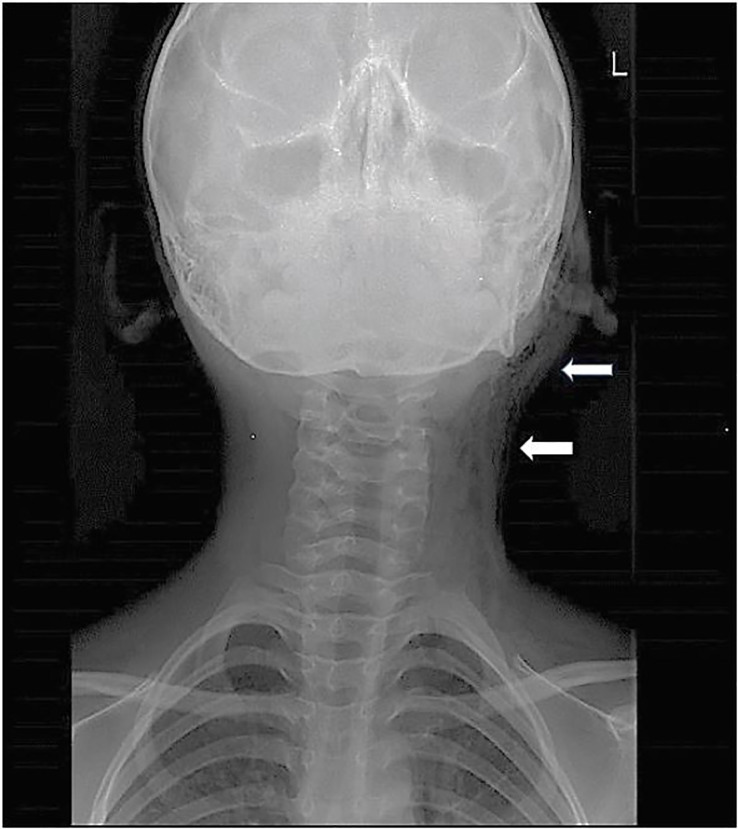
Anterior-posterior radiograph of the neck demonstrating extensive left-sided cervicofacial subcutaneous emphysema (arrows).

**Image 3:**
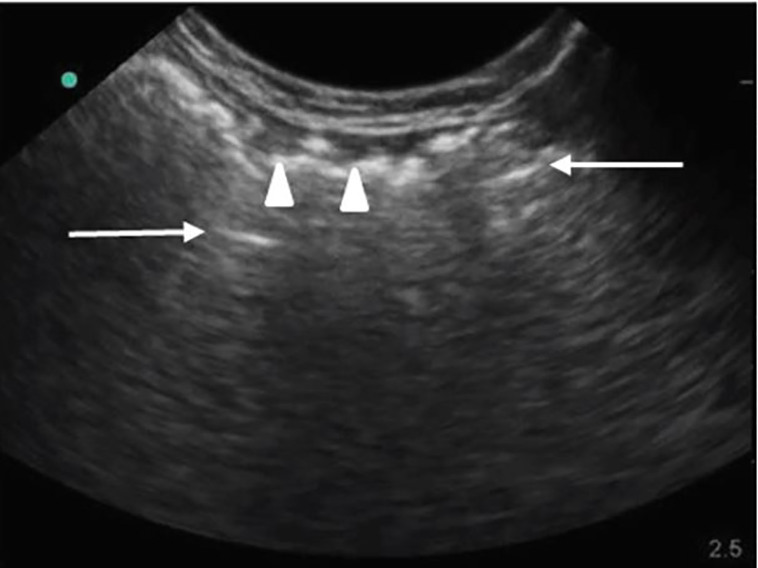
Point-of-care ultrasound of the neck done by the emergency physician, showing hyperechoic soft-tissue emphysema (arrowheads) with posterior acoustic shadowing and reverberation artifacts (arrows).

Radiographs of the neck (Image 2) and chest showed extensive subcutaneous emphysema involving the left side of the face and neck but no evidence of pneumomediastinum or pneumothorax. Point-of-care ultrasound (POCUS) performed by the emergency physician showed the presence of soft-tissue emphysema as hyperechoic areas (air pockets) with acoustic shadowing (Image 3).

He was diagnosed with left pneumoparotitis with cervicofacial subcutaneous emphysema for which otorhinolaryngology was consulted. Endoscopic nasolaryngoscopy revealed normal nasal septum, normal turbinates with no pus or polyps, and normal adenoids. The larynx and hypo-pharynx looked normal with no evidence of trauma or infection, normal mobile vocal cords, no foreign bodies, and no masses. All hematology, infective markers and biochemical (urea, creatinine and electrolytes) investigations were essentially normal. The patient was admitted for further evaluation and inpatient monitoring for progression and complications.

**CPC-EM Capsule**

What do we already know about this clinical entity?

Pneumoparotitis with cervicofacial emphysema is an uncommon presentation of cheek swelling in children. On progression, it may lead to air leak syndromes, infection and thromboembolism.

What makes this presentation of disease reportable?

This uncommon pediatric diagnosis, occurring spontaneously in a previously well child with no apparent risk factors, was made using point-of-care ultrasound (POCUS) and radiographs.

What is the major learning point?

Pneumoparotitis with cervicofacial subcutaneous emphysema is a rare but important differential diagnosis for facial swelling, with potential for serious complications.

How might this improve emergency medicine practice?

POCUS and radiographs are simple and useful clinical adjuncts that can be used to facilitate a quick and early diagnosis for this rare condition in children.

He was treated empirically with antibiotics, given analgesia and was put on supplemental oxygen with a non-rebreather mask to provide 100% FiO_2_. Despite appropriate management, his condition progressed with bilateral cervicofacial involvement. In view of this, a contrast-enhanced computed tomography (CT) of the neck was performed the following day, which showed bilateral extensive subcutaneous emphysema tracking proximally from both temporal regions to the anterior mediastinum distally. Clinically, he remained stable with no evidence of hemodynamic or respiratory compromise. After 72 hours, his condition started to stabilize. All blood cultures, blood and inflammatory markers were unremarkable. He showed significant clinical improvement and was discharged well after five days of hsopitalization. His pneumoparotitis and cervicofacial subcutaneous emphysema had completely resolved on review at the specialist outpatient clinic five days post-discharge.

## DISCUSSION

Due to a lack of a leading history, idiopathic pneumoparotitis and cervicofacial subcutaneous emphysema in children may present as diagnostic challenges to emergency physicians.^1^,^2^ Differentials that should be considered include infections (such as mumps, cellulitis, and parotid abscess) and other causes (such as haemorrhage, angioedema and Melkersson-Rosenthal syndrome).^1^–^3^ The clinical features of pneumoparoitis and cervicofacial emphysema can have immediate manifestations including local swelling, crepitus and discomfort. Local erythema, tenderness, dysphagia, dyspnoea and trismus may also develop. It is important to exclude potentially life-threatening causes of cervicofacial subcutaneous emphysema such as oesophageal perforations, necrotising soft tissue and parotid infections, retro-pharyngeal abscess and mediastinitis. Progressive complications can occur and may lead to significant air leak syndromes such as upper airway obstruction, pneumomediastinum or overt pneumothoraces.

Other reported potential complications include air embolism and pulmonary embolism.^5^ When air gets introduced into the fascial planes in the head and neck, which contains loose connective tissue between the layers of muscles and other structures, the air takes the path of least resistance. The air then subsequently enters the retropharyngeal space. From there, the air can migrate into the “danger space” of Grodinsky and Holyoke, which is in direct communication with the posterior mediastinum. The collection of air in this space can compress the venous trunk resulting in cardiac failure or tracheal compression causing asphyxia.

Another rare complication is the occurrence of air embolism, which is a result of entry of air into an open vessel due to erosion of the vessel wall. The air thus entering into the vessel can reach the right side of the heart followed by entry into the pulmonary vasculature causing pulmonary embolism.^5^ In 20–30% of the pediatric population in which patent foramen ovale is present, air can also enter coronaries and cerebral vasculature causing fatal complications. Damage to the optic nerve can occur as a result of progressive accumulation of air around the optic foramen.

The presence of presence of subcutaneous air on plain radiograph and CT are very specific for the diagnosis of subcutaneous emphysema. CT is also capable of distinguishing emphysema from necrotising fasciitis caused by gas-forming organisms. However, we feel that CT neck should not be routinely performed for initial diagnosis in uncomplicated cases; due to radiation exposure to the thyroid gland and other associated long-term radiation risks in children. For our patient, this was performed later as an inpatient in view of the initial progression of his condition despite appropriate management. We felt that POCUS and radiographs were useful adjuncts that facilitated an early diagnosis.

Provision of supplemental oxygen will help hasten the resorption of the air accumulated in the emphysematous cavity by replacing the nitrogen with oxygen. Initial supportive care includes analgesia and alleviating aggravating factors such as cough with antitussive medication, preventing retching and vomiting with anti-emetics, and avoiding straining at defecation by using stool softeners.^1^–^4^ However, for our patient, his condition was unprovoked. Prophylactic broad spectrum antibiotics have been advocated by some specialists in preventing the development of post-resolution purulence in the emphysematous cavity.^4^,^5^ Complete resolution of signs and symptoms can be usually expected in 7–10 days.^1^–^4^

## CONCLUSION

Idiopathic pneumoparotitis with cervicofacial emphysema is a rare but important condition for clinicians to recognize and diagnose early. The use of clinical adjuncts such as POCUS and radiographs may be useful to facilitate an early diagnosis.
